# Interpersonal Distress as a Covariate of Mental Health in Depression: A Multilevel Meta‐Analysis

**DOI:** 10.1002/cpp.70143

**Published:** 2025-08-18

**Authors:** Juan Martín Gómez Penedo, Malenka Areas, Cristoph Flückiger

**Affiliations:** ^1^ University of Kassel Germany Kassel Germany; ^2^ Osnabrück University Osnabrück Germany; ^3^ Universidad de Buenos Aires and CONICET Buenos Aires Argentina

**Keywords:** depression, interpersonal distress, mental health, meta‐analysis, systematic review

## Abstract

Although empirical studies have shown that interpersonal distress is related to mental health indicators in depression, there are no previous meta‐analyses evaluating this association. We performed a systematic review and meta‐analytic study to estimate the association between interpersonal distress and non‐relational mental health indicators (NR‐MHI) in depression. Following PRISMA criteria, we performed a systematic search on PubMed and PsycINFO. We conducted multilevel meta‐analytic (i.e., random‐effects) models to estimate a pooled correlation coefficient representing the strength of the association between interpersonal distress and NR‐MHI. Thirty‐eight studies (reporting 88 effect sizes) met inclusion criteria. The models showed a significant correlation between interpersonal distress and NR‐MHI (*r* = 0.41, *p* < 0.001), with moderate heterogeneity, Q(87) = 710.38, *p* < 0.001. A funnel plot did not present evidence of publication bias. We found no significant moderation effects of specific depressive diagnoses, type of study or levels of interpersonal distress. This study is the first to report a meta‐analytic synthesis of the association between interpersonal distress and NR‐MHI in depression, showing that in individuals with depression, NR‐MHI was positively associated with interpersonal distress, presenting a medium‐to‐large pooled effect size.

## Summary

- Interpersonal distress is significantly associated with negative mental health indicators in individuals with depression, with a medium‐to‐large effect size.
- Assessing interpersonal problems in patients with depression may provide additional insight into symptom severity and co‐occurring psychological difficulties.
- Although causality cannot be inferred, these findings support the inclusion of interpersonal functioning in comprehensive models of depression assessment and treatment.

## 1 Introduction

Depressive disorders have a high and increasing prevalence worldwide, affecting approximately 5% of the adult population (G.B.D. 2015 Disease and Injury Incidence and Prevalence Collaborators 2016; World Health Organization [WHO] 2023). It has been estimated that each year, the number of individuals affected by depressive disorders exceeds 280 million (WHO 2023). Empirical findings have shown that depressive disorders are significant contributors to both global disease rates and financial burdens (The Lancet Global Health 2020; Yang et al. 2021), being leading causes of years lived with disability in the world (Pan American Health Organization 2018).

Although psychopathology in general and depressive disorders, in particular, are predominantly conceptualized as discrete diagnostic categories of mental disorders, more recent models examine psychopathology as continuous dimensions of maladaptive behaviours, emotions and cognitions and their interplay (Lahey et al. 2021). Several theories emphasize the potential relevance of interpersonal distress in depression. For example, Lewinsohn's social reinforcement theory (Lewinsohn 1974) highlighted the low levels or lack of positive reinforcements in the interpersonal environment of depressed individuals (e.g., whereas the environment does not provide reinforcement, or the individual does not have the required social skills to get reinforcement, or the individual is unable to detect and enjoy social reinforcement). Coyne (1976) also postulated that the environmental response to people with depression has a fundamental role in the development and maintenance of the condition. From this perspective, depression results from a disruption in the interpersonal environment from which the person receives validation and support. Additionally, this theory states that the depressive behaviours in terms induce negative feelings in their interactants, resulting in rejection and a further loss of support and validation from the environment, enhancing/maintaining depressive symptoms and behaviours. Moreover, recent studies showed that the perception of an individual's intimate, relational or collective loneliness may enhance the risk for depression (e.g., Cacioppo et al. 2015).

Within that wider framework, interpersonal dysfunctions (measured as the overall mean of the inventory of interpersonal problems) have been specifically hypothesized as a fundamental contributor to psychopathology (Horowitz 2004; Hopwood et al. 2023). Particularly in depression, empirical research has shown that interpersonal distress is associated with depressive severity (Barrett and Barber 2007) and other mental health indicators such as quality of life (McEvoy et al. 2013), self‐criticism (Dinger et al. 2013) and self‐esteem (Sowislo and Orth 2013).

Some systematic reviews and meta‐analyses have also associated depression with interpersonally related variables such as social support (Edwards et al. 2020) and loneliness (Erzen and Çikrikci 2018). Furthermore, a recent multilevel meta‐analysis demonstrated significant effects of baseline interpersonal distress on psychotherapy outcomes for depressive and anxiety disorders (Gómez Penedo and Flückiger 2023). However, despite the relevance of depression in mental health and the evidence supporting the association of relational variables with depression severity, there is no previous meta‐analytic synthesis evaluating specifically how interpersonal distress is related to negative mental health indicators in depression. A better evidence‐based understanding of the role of interpersonal distress in depression might help further understand the etiopathogenesis of the condition and enhance the development of interventions that could address it.

## 2 Current Systematic Review and Meta‐Analysis

In this study, following Preferred Reporting Items for Systematic Reviews and Meta‐Analyses (PRISMA) guidelines (Page et al. 2021), we performed a systematic review and meta‐analytic synthesis to estimate the association between interpersonal distress and non‐relational mental health indicators (NR‐MHI) in individuals diagnosed with depression. We expected that interpersonal distress would be associated with poorer mental health indicators in depression, presenting at least a medium effect size.

## 3 Methods

### 3.1 Meta‐Analytic Search Strategy, Study Selection and Data Collection

The aim and methods of the paper were pre‐documented on the Open Science Foundation (https://osf.io/7w85m/?view_only=ba20db7eec7649e7b89db23df0865498). In this study, we conducted a systematic search through PsycINFO and PubMed databases for all the published studies until April 2025. The search string included the terms ‘interpersonal problems’, ‘interpersonal distress’, ‘interpersonal dysfunction’ and ‘depression’, ‘dysthymia’ or ‘depressive’ and included peer‐reviewed publications and dissertations. Papers eligibility criteria included: (1) empirical studies having a sample being either (a) diagnosed with depressive disorders or (b) from a general clinical population where at least 70% of the participants present depressive disorders (i.e., implying that the vast majority of the participants were diagnosed with a depressive disorder); (2) focusing on an adult population (i.e., x̄ age > 18 years); (3) presenting quantitative data; (4) measuring at least NR‐MHI; (5) using a measure of interpersonal distress (e.g., Inventory of Interpersonal Problems; grosse Holtforth et al. 2006; Horowitz et al. 2000); (6) providing an association between interpersonal distress and NR‐MHI; (7) having enough quantitative information to calculate effect sizes. We included studies in English, German and Italian. Single‐case studies were not included in the meta‐analysis. Within NR‐MHI, we included measures of overall psychological distress, depression severity, anxiety severity, functioning, well‐being, pathological personality traits, pathological beliefs, self‐esteem and comorbidity.

In summary, our systematic search included empirical studies with adult samples diagnosed with depressive disorders or from a general clinical population, with the majority of patients having depressive disorders, which reported quantitative information regarding the contemporaneous association between interpersonal distress and at least one NR‐MHI.

**FIGURE 1:**
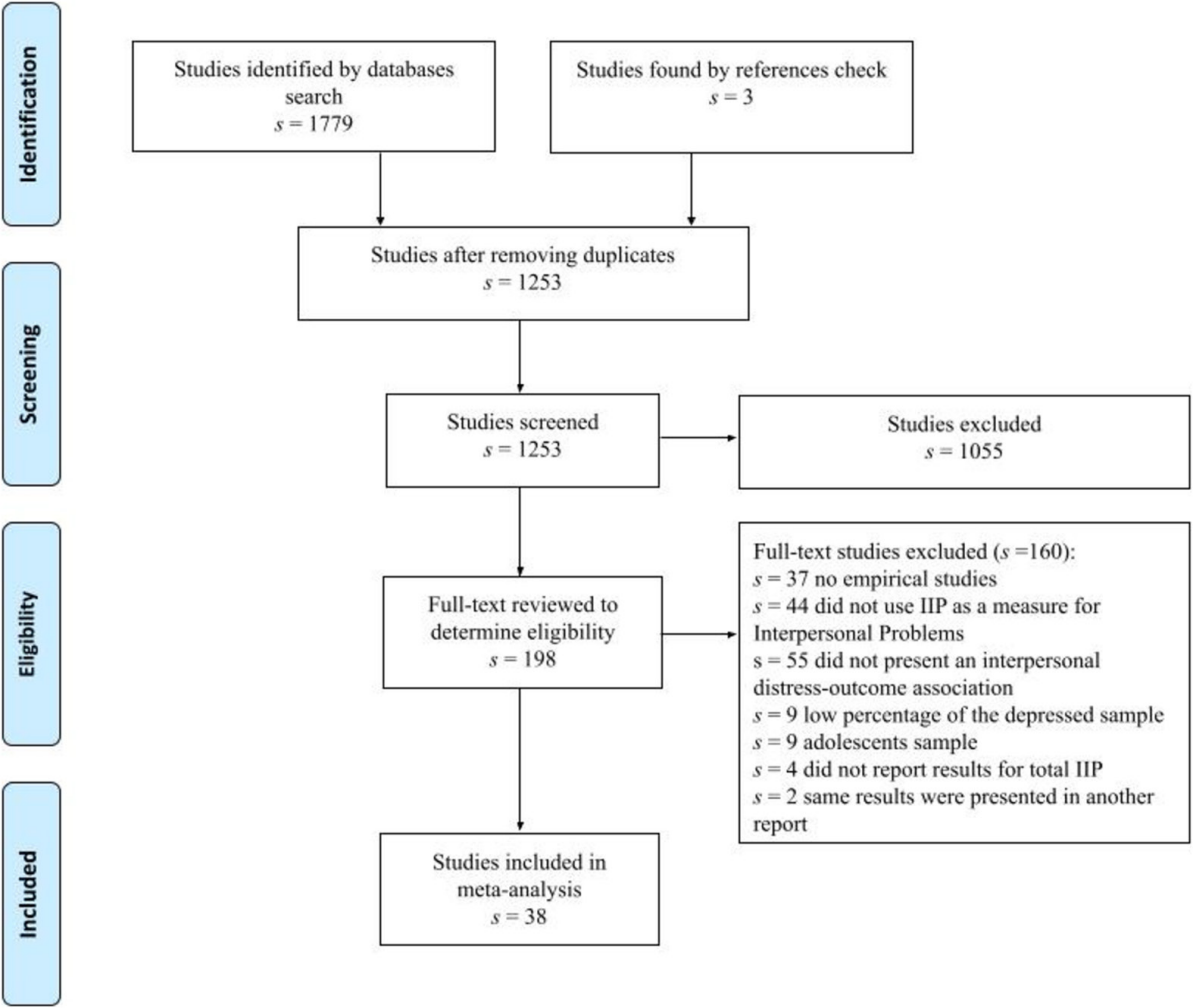
Flow diagram of the studies included in the meta‐analysis based on PRISMA criteria (Page et al. 2021).

The flowchart in Figure 1 provides an overview of the extraction procedure. The search yielded 1782 records. Three additional papers were included based on the reference lists of other published papers and systematic reviews.

For the first screening, two researchers (first and second author of the paper) independently reviewed all the study titles and abstracts to determine eligibility. Then, we obtained full texts for the papers that were potentially eligible, to finally define their eligibility (82% agreement). In the case of disagreement, studies were included in the further meta‐analytic steps. The extraction of effect sizes was performed also by the two researchers based on the full texts (90% agreement). Any disagreements at all stages of the process were discussed with the paper's last author, as a supervisor, and addressed through consensus. After screening all full texts, we concluded that 36 studies met the criteria for eligibility and were included in the meta‐analytic analyses.

### 3.2 Data Analysis

**FIGURE 2:**
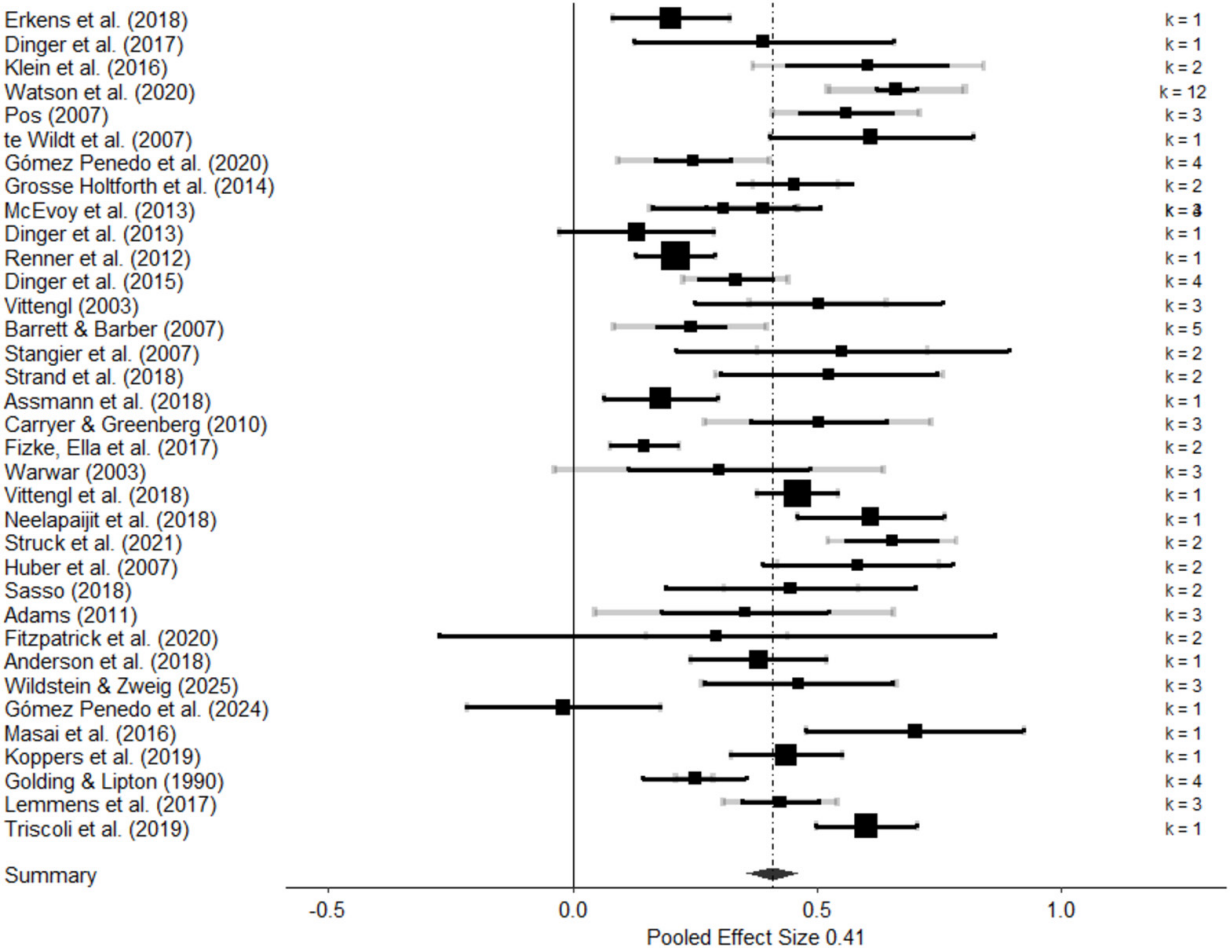
Forest plot of the pooled correlational (*r*) effect size. The grey confidence interval illustrates the within‐effect sizes variability based on the sampling variance (Level 1). The black confidence interval represents the variability at the within‐study level (Level 2).

For the analyses, we used the correlation coefficient, Pearson's *r*, as the effect size measure. Other effect size measures reported (e.g., mean comparisons) were transformed into *r*s. All effect sizes were coded towards higher interpersonal problems—worse mental health indicators. In the case of multiple effect sizes reported on a single study, all effect sizes were coded and included. Effect sizes reported for different subgroups within the same manuscript (e.g., different populations, diagnoses, etc.) were treated as independent samples, and therefore coded with a different identifier. To illustrate the distribution of the effect sizes, we used a multilevel forest plot (Figure 2) (Fernández‐Castilla et al. 2020).

All the analyses were conducted using Fisher's *z* transformation of the correlation coefficient, considering that the effect sizes may not be normally distributed (see Borenstein et al. 2009). Then, the results were transformed back into *r* coefficients to enhance interpretability. The magnitudes of the correlations were interpreted as follows (Cohen 1992): 0.10 small effect size, 0.30 medium effect size and 0.50 large effect size.

For the meta‐analytic summary, correlational multilevel meta‐analyses were performed using random‐effects models, addressing within‐effect size, between‐effect size and between‐study variability with a three‐level model structure (Assink and Wibbelink 2016). To estimate the multilevel meta‐analytic models, we used the *R* package *metafor* (Viechtbauer 2010). In computing the overall effect (with no moderators), independent effects were weighted by their sample size. Heterogeneity was assessed with the Q and *I* ^2^ statistics.

Furthermore, as part of the sensitivity analysis, we examined the following moderators of interpersonal distress and mental health indicators association:

- Depressive‐specific versus non‐depressive specific sample: if (1) inclusion criteria of a study required a depression diagnosis or (0) the large majority (70%) of a natural sample indicated a depression diagnosis.
- Treatment study or not: if (1) a study was a trial (receiving, e.g., psychotherapy) or (0) a non‐interventional study.
- Types of outcome measures with depression severity as the reference category. All effect sizes were classified into the following outcome domains: (1) comorbidity comparison (e.g., comparing depression with other clinical conditions), (2) healthy control comparison, (3) anxiety measures, (4) general symptomatology measures, (5) personality measures and (6) self‐esteem measures. Assessing whether the association between interpersonal distress and mental health indicators differed significantly across these outcome types, in comparison with depression severity outcomes.

A funnel diagram, which plots the standard error (vertical axis) and effect sizes of each study (horizontal axis) was also created and analysed to identify publication bias (Fernández‐Castilla et al. 2020). Finally, the effects of outliers were checked using standard formulas grounded on the interquartile range (IQR; Walfish 2006). Cases that were 1.5 IQR below the first quartile or above the third quartile were considered outliers.

## 4 Results

### 4.1 Primary Study and Effect Size Characteristics

The 38 studies that met inclusion criteria provided data from *N* = 8132 patients, including 88 effect sizes of the contemporaneous association between interpersonal distress and NR‐MHI. Descriptive data for the included studies are presented at Table 1. All studies were conducted either in North America or Europe, with the exception of two studies conducted in Asia and one in Australia. Although the majority of studies received peer review, we also detected and included studies without peer review (i.e., Adams 2010; Bernholtz 2016; Pos 2007; Sasso 2018; Warwar 2003). Furthermore, details of the effect sizes included in this meta‐analysis are presented in Table 2.

**TABLE 1:** Main characteristics of the studies meeting inclusion criteria for the meta‐analysis (*s* = 38).

Authors	Country	ID‐measure	Peer reviewed	Mean age	Female %	Diagnoses or proportion of depression	Comorbid substance abuse	Comorbid anxiety disorder	Comorbid personality disorders	Personality disorders %	Psychotherapy sample
Adams (2010)	Canada	IIP‐127	No	44.1	71%	MDD	No	No	No	—	Yes
Altenstein‐Yamanaka et al. (2017)(**)	Switzerland	IIP‐32	Yes	40.7	56.3%	MDD	No	Yes	Yes	23.6%	Yes
Anderson et al. (2018)	USA	IIP‐64	Yes	51.9	38%	MDD	No	No	No	—	Yes
Assmann et al. (2018)	Germany	IIP‐64	Yes	44.9	66%	MDD	No	Yes	No	—	Yes
Barrett and Barber (2007)	USA	IIP‐C	Yes	37.8	60%	MDD	No	Yes	Yes	61%	No
Bernholtz (2016) (*)	Canada	IIP‐127	No	41.52	67%	MDD	No	No	No	—	Yes
Carryer and Greenberg (2010)	Canada	IIP‐127	Yes	39.5	63%	DD	No	Yes	Yes	21%	Yes
Dinger et al. (2015)	Germany	IIP‐64	Yes	36.9	63.6%	MDD	No	No	No	—	Yes
Dinger et al. (2017)	Germany	IIP‐64	Yes	35.5	50%	DD	No	Yes	Yes	30%	Yes
Dinger et al. (2013)	Germany	IIP‐64	Yes	37.5	60.9%	MDD	No	No	No	—	Yes
Erkens et al. (2018)	Germany	IIP‐64	Yes	44.6	64.8%	DD	No	No	Yes	52.2%	Yes
Fitzpatrick et al. (2020)	USA	IIP‐32	Yes	31.7	60%	MDD	No	No	No	—	Yes
Fizke et al. (2017)	Germany	IIP‐64	Yes	38	71.3%	DD	Yes	Yes	Yes	25%	Yes
Golding and Lipton (1990)	USA	CES‐D	Yes	39.35	50.25%	MDD	No	No	No	—	No
Gómez Penedo et al. (2025)	Germany	IIP‐32	Yes	31.2	57%	DD	No	Yes	Yes	74%	Yes
Gómez Penedo et al. (2020)(**)	Switzerland	IIP‐32	Yes	40.8	55.3%	MDD	No	Yes	Yes	27.7%	Yes
grosse Holtforth et al. (2014)	Switzerland	IIP‐C	Yes	37.8	55.7%	DD	No	Yes	No	—	Yes
Huber et al. (2007)	Germany	IIP‐D	Yes	33	64%	DD	No	No	Yes	32%	Yes
Klein et al. (2016)	Germany	IIP‐64	Yes	CD: 49.8 ED: 35.6	73%	CD&EP	Yes	Yes	No	—	No
Koppers et al. (2019)	Netherlands	CES‐D	Yes	38.91	68.3%	DD	No	Yes	Yes	67.9%	Yes
Lemmens et al. (2017)	Netherlands	IIP‐64	Yes	41.2	66.2%	MDD	No	No	No	—	Yes
Masai et al. (2016)	Japan	CES‐D		59.4	66.6%	MDD	No	No	No	—	Yes
McEvoy et al. (2013)	Australia	IIP‐32	Yes	36.4	65%	MDD: 76.8%	No	Yes	No	—	No
McEvoy et al. (2013)	Australia	IIP‐32	Yes	38.56	68.1%	MDD: 88.2%	No	Yes	No	—	Yes
Neelapaijit et al. (2018)	Thailand	IIP‐32	Yes	38.3	60.3%	DD: 76.5%	No	No	No	—	No
Pos (2007)	Canada	IIP‐127	No	39.93	66%	DD	No	Yes	Yes	34%	Yes
Renner et al. (2012)	USA	IIP‐C	Yes	42.2	67.5%	MDD	No	No	No	—	Yes
Sasso (2018)	USA	IIP‐32	No	32	59%	MDD	No	Yes	No	—	Yes
Stangier et al. (2007)	Germany	IIP‐127	Yes	41.3	62%	MDD	Yes	No	Yes	45%	No
Strand et al. (2018)	Norway	IIP‐64	Yes	33.7	59%	DD	No	Yes	Yes	33%	Yes
Struck et al. (2021)	Germany	IIP‐32	Yes	ED:39.9 PDD:41.6	ED:53.8% PDD:50%	ED&PDD	No	No	No	—	No
te Wildt et al. (2007)	Germany	IIP‐D	Yes	29.4	22%	DD	No	No	No	—	No
Triscoli et al. (2019)	Sweden	IIP‐64	Yes	35.44	78%	DD	No	Yes	No	—	No
Vittengl et al. (2003)	USA	IIP‐C	Yes	42.7	74.6%	MDD	No	No	No	—	Yes
Vittengl et al. (2018)	USA	IIP‐127	Yes	43.3	67.8%	DD	No	No	No	—	No
Warwar (2003)	Canada	IIP‐127	No	37	60%	DD	No	No	Yes	40%	Yes
Watson et al. (2020) (*)	Canada	IIP‐127	Yes	41.52	66.7%	DD	No	No	Yes	51.5%	Yes
Wildstein and Zweig (2025)	USA	IIP‐25	Yes	67.60	45.2%	DD	No	No	No	—	Yes

*Note:* Papers with (*) and (**) were based on the same dataset but reported different outcomes.

Abbreviations: % = percentage; BPD, bipolar disorder; CD, chronic depression; DD, depressive disorder; ED, episodic depression; ID, interpersonal distress; MDD, major depressive disorder; PDD, persistent depression disorder.

**TABLE 2:** Characteristics of the effect size reported (*k* = 88) within the studies of the meta‐analysis (*s* = 38).

Author, year	Outcome	Post‐treatment	*n* patients	Reported ES	*r* ^a^	Sample variances
Adams (2010)	Depression severity (BDI)	No	34	*r* = 0.25	0.25	0.027
Adams (2010)	Self‐esteem (RSE)	No	34	*r* = −0.45	0.45	0.019
Adams (2010)	Symptoms severity (SCL‐90)	No	34	*r* = 0.32	0.32	0.024
Altenstein‐Yamanaka et al. (2017)(**)	Depression severity (BDI)	No	144	*r* = 0.23	0.23	0.006
Altenstein‐Yamanaka et al. (2017)	Depression severity (IDS)	No	144	*r* = 0.22	0.22	0.006
Altenstein‐Yamanaka et al. (2017)	Symptoms severity (SCL‐9)	No	144	*r* = 0.31	0.31	0.006
Anderson et al. (2018)	Depression severity (BDI)	Yes	147	*r* = 0.38	0.38	0.005
Assmann et al. (2018)	COM (*n* = 174) or not COM ad (*n* = 88)	No	262	ad: 15.84 (3.8), no ad: 14.43 (3.58)	0.18	0.004
Barrett and Barber (2007)	Depression severity (BDI)	No	141	*r* = 0.29	0.29	0.006
Barrett and Barber (2007)	Depression severity (HRSD)	No	141	*r* = 0.23	0.23	0.006
Barrett and Barber (2007)	COM (*n* = 86) or not COM axis I (*n* = 55)	No	141	COM.: 1.4 (1), not COM.: 0.96 (1.1)	0.20	0.007
Barrett and Barber (2007)	COM (*n* = 67) or not COM axis II (*n* = 74)	No	141	COM.: 1.6 (0.96), not COM.: 0.93 (1.1)	0.31	0.006
Barrett and Barber (2007)	COM (*n* = 23) or not COM GAD (*n* = 118)	No	141	COM.: 1.4 (1), not COM.: 0.92 (1.1)	0.16	0.007
Bernholtz (2016) (*)	Depression severity (BDI)	No	66	*r* = 0.66	0.66	0.005
Bernholtz (2016)	Self‐esteem (RSE)	No	66	*r* = −0.60	0.60	0.007
Bernholtz (2016)	Dysfunctional attitudes (DAS)	No	66	*r* = 0.67	0.67	0.005
Bernholtz (2016)	Symptoms severity (SCL‐90)	No	66	*r* = 0.62	0.62	0.006
Bernholtz (2016)	Reflective coping style (PF‐SOC)	No	66	*r* = −0.47	0.47	0.010
Bernholtz (2016)	Suppressive coping style (PF‐SOC)	No	66	*r* = 0.64	0.64	0.006
Bernholtz (2016)	Reactive coping style (PF‐SOC)	No	66	*r* = 0.58	0.58	0.007
Carryer and Greenberg (2010)	Depression severity (BDI)	No	38	*r* = 0.43	0.43	0.018
Carryer and Greenberg (2010)	Symptoms severity (SCL‐90)	No	38	*r* = 0.53	0.53	0.014
Carryer and Greenberg (2010)	Self‐esteem (RSE)	No	38	*r* = −0.53	0.53	0.014
Dinger et al. (2015)	Dependency (DEQ)	No	273	*r* = 0.37	0.37	0.005
Dinger et al. (2015)	Self‐criticism (DEQ)	No	273	*r* = 0.40	0.40	0.005
Dinger et al. (2015)	Depression severity (HRSD)	No	276	*r* = 0.23	0.23	0.006
Dinger et al. (2015)	Depression severity (BDI)	No	195	*r* = 0.32	0.32	0.005
Dinger et al. (2013)	Depression severity (HRSD)	No	151	*F*_(1, 150.2)_ = 3.40	0.13	0.006
Dinger et al. (2017)	Self‐esteem (RSE)	No	40	*r* = −0.39	0.39	0.018
Erkens et al. (2018)	COM (*n* = 95) or not COM PD (*n* = 152)	No	247	PD: 15.85 (3.2), no PD: 14.34 (3.78)	0.20	0.004
Fitzpatrick et al. (2020)	Depression severity (BDI)	No	126	*r* = 0.00	0	0.008
Fitzpatrick et al. (2020)	PD traits (PID‐5‐BF)	No	126	*r* = 0.58	0.58	0.004
Fizke et al. (2017)	COM (*n* = 179) or not COM PD (*n* = 536)	No	715	PD: 1.90 (0.47), no PD: 1.68 (0.53)	0.18	0.001
Fizke et al. (2017)	COM (*n* = 179) or not COM PD (*n* = 536)	Yes	715	PD: 1.61 (0.59), no PD: 1.47 (0.52)	0.11	0.001
Golding and Lipton (1990)	CES‐D IP and depression	No	2244	*r* = 0.12	0.12	0.004
Golding and Lipton (1990)	CES‐D IP and negative affect	No	2244	*r* = 0.34	0.34	0.003
Golding and Lipton (1990)	CES‐D IP and somatic disturbance	No	2244	*r* = 0.34	0.34	0.003
Golding and Lipton (1990)	CES‐D IP and lack of positive affect	No	2244	*r* = 0.20	0.20	0.004
Gómez Penedo et al. (2020)(**)	Well‐being (WHO‐5)	No	141	*r* = −0.22	0.22	0.011
Gómez Penedo et al. (2025)	Depression severity (HRSD)	No	100	*r* = −0.02	0.02	0.010
grosse Holtforth et al. (2014)	Depression severity (BDI)	No	292	*r* = 0.39	0.39	0.002
grosse Holtforth et al. (2014)	Symptoms severity (BSI‐GSI)	No	359	*r* = 0.51	0.51	0.002
Huber et al. (2007)	Inhibitedness (FPI‐R)	No	63	*r* = 0.47	0.47	0.010
Huber et al. (2007)	Aggression turning against self (FKBS)	No	63	*r* = 0.67	0.67	0.005
Klein et al. (2016)	CD (*n* = 15) or HC (*n* = 15)	No	30	CD: 15.02 (3.94), HC: 8.52 (3.63)	0.65	0.012
Klein et al. (2016)	ED (*n* = 15) or HC (*n* = 15)	No	30	ED: 14.18 (5.35), HC: 8.52 (3.63)	0.53	0.018
Koppers et al. (2019)	COM PD (*n* = 133) or not COM (*n* = 63)	No	196	PD: 21.92 (5.16), no PD: 16.41 (6.18)	0.44	0.003
Lemmens et al. (2017)	Depression severity (BDI)—pre‐treatment	No	182	*r* = 0.48	0.48	0.003
Lemmens et al. (2017)	Depression severity (BDI)—mid	No	182	*r* = 0.44	0.44	0.004
Lemmens et al. (2017)	Depression severity (BDI)—post‐treatment	Yes	182	*r* = 0.34	0.34	0.004
Masai et al. (2016)	CES‐D IP with GAF	No	21	*r* = −0.70	0.70	0.010
McEvoy et al. (2013)	Depression severity (BDI)	No	457	*r* = 0.47	0.47	0.001
McEvoy et al. (2013)	Anxiety severity (BAI)	No	450	*r* = 0.27	0.27	0.002
McEvoy et al. (2013)	Quality of Life (Q‐LES‐Q)	No	486	*r* = −0.42	0.42	0.001
McEvoy et al. (2013)	Depression severity (BDI)	No	144	*r* = 0.34	0.34	0.005
McEvoy et al. (2013)	Anxiety severity (BAI)	No	144	*r* = 0.16	0.16	0.007
McEvoy et al. (2013)	Depression severity (CCLD)	No	144	*r* = 0.49	0.49	0.004
McEvoy et al. (2013)	Quality of life (Q‐LES‐Q)	No	144	*r* = −0.22	0.22	0.006
Neelapaijit et al. (2018)	Pathogenic beliefs (PBS)	No	68	*r* = 0.61	0.61	0.006
Pos (2007)	Depression severity (BDI)	No	74	*r* = 0.43	0.43	0.009
Pos (2007)	Symptoms severity (SCL‐90)	No	74	*r* = 0.59	0.59	0.006
Pos (2007)	Self‐esteem (RSE)	No	74	*r* = −0.61	0.61	0.005
Renner et al. (2012)	Depression severity (HRSD)	No	523	*F*_(1, 521.12)_ = 24.82	0.21	0.002
Sasso (2018)	Depression severity (BDI)	No	126	*r* = 0.31	0.31	0.007
Sasso (2018)	PD traits (PID‐5‐BF)	No	126	*r* = 0.57	0.57	0.004
Stangier et al. (2007)	Depression (*n* = 53) or HC (*n* = 24)	No	77	D: 122 (31.2), HC: 53.2 (33.4)	0.71	0.003
Stangier et al. (2007)	Intrapersonal conflict (ICT)	No	53	*r* = 0.36	0.36	0.015
Strand et al. (2018)	Depression severity (BDI)	No	39	*r* = 0.39	0.39	0.019
Strand et al. (2018)	Depression severity (BDI)	Yes	39	*r* = 0.62	0.62	0.010
Struck et al. (2021)	ED (*n* = 38) or HC (*n* = 39)	No	77	D: 1.9 (0.44), HC: 1.2 (0.5)	0.60	0.005
Struck et al. (2021)	PDD (*n* = 34) or HC (*n* = 39)	No	73	PDD: 2.07 (0.41), HC: 1.2 (0.5)	0.69	0.004
te Wildt et al. (2007)	Depression (*n* = 18) or HC (*n* = 18)	No	36	D:1.41 (0.61), HC: 0.60 (0.43)	0.61	0.011
Triscoli et al. (2019)	Non depressed (*n* = 77) or depressed (*n* = 70)	No	144	D: 89.4 (20.9), Non D: 57.4 (21.8)	0.60	0.007
Vittengl et al. (2003)	Negative temperament (SNAP)	Yes	102	*r* = 0.70	0.70	0.003
Vittengl et al. (2003)	Positive Temperament (SNAP)	Yes	102	*r* = −0.53	0.53	0.005
Vittengl et al. (2003)	Disinhibition (SNAP)	Yes	102	*r* = 0.25	0.25	0.009
Vittengl et al. (2018)	Depression severity (BDI and IDS‐SR)	No	351	*r* = 0.46	0.46	0.002
Warwar (2003)	Depression severity (BDI)	No	31	*r* = 0.16	0.16	0.031
Warwar (2003)	Symptoms severity (SCL‐90)	No	31	*r* = 0.24	0.24	0.029
Warwar (2003)	Self‐esteem (RSE)	No	31	*r* = −0.44	0.44	0.021
Watson et al. (2020) (*)	Depression severity (BDI)	Yes	66	Rho = 0.74	0.74	0.003
Watson et al. (2020)	Dysfunctional attitudes (DAS)	Yes	66	Rho = 0.71	0.71	0.004
Watson et al. (2020)	Self‐criticism (DES)	Yes	66	Rho = 0.75	0.75	0.003
Watson et al. (2020)	Neediness (DES)	Yes	66	Rho = 0.57	0.57	0.007
Watson et al. (2020)	Self‐esteem (RSE)	Yes	66	Rho = −0.68	0.68	0.004
Wildstein and Zweig (2025)	NEO‐FFI neuroticism	No	62	*r* = 0.61	0.61	0.006
Wildstein and Zweig (2025)	NEO‐FFI agreeableness	No	62	*r* = −0.28	0.28	0.014
Wildstein and Zweig (2025)	Geriatric depression scale	No	62	*r* = 0.45	0.45	0.010

Abbreviations: AD, anxiety disorder; CD, chronic depression; COM, comorbid; D, depression; ED, episodic depression; GAD, generalized anxiety disorder; GAF, global assessment of functioning; HC, healthy control; IP, interpersonal problems; PD, personality disorder; PDD, persistent depressive disorder.

^a^ The final *r* was inverted to match the correct direction.

### 4.2 Interpersonal Problems and NR‐MHI in Depression

**FIGURE 3:**
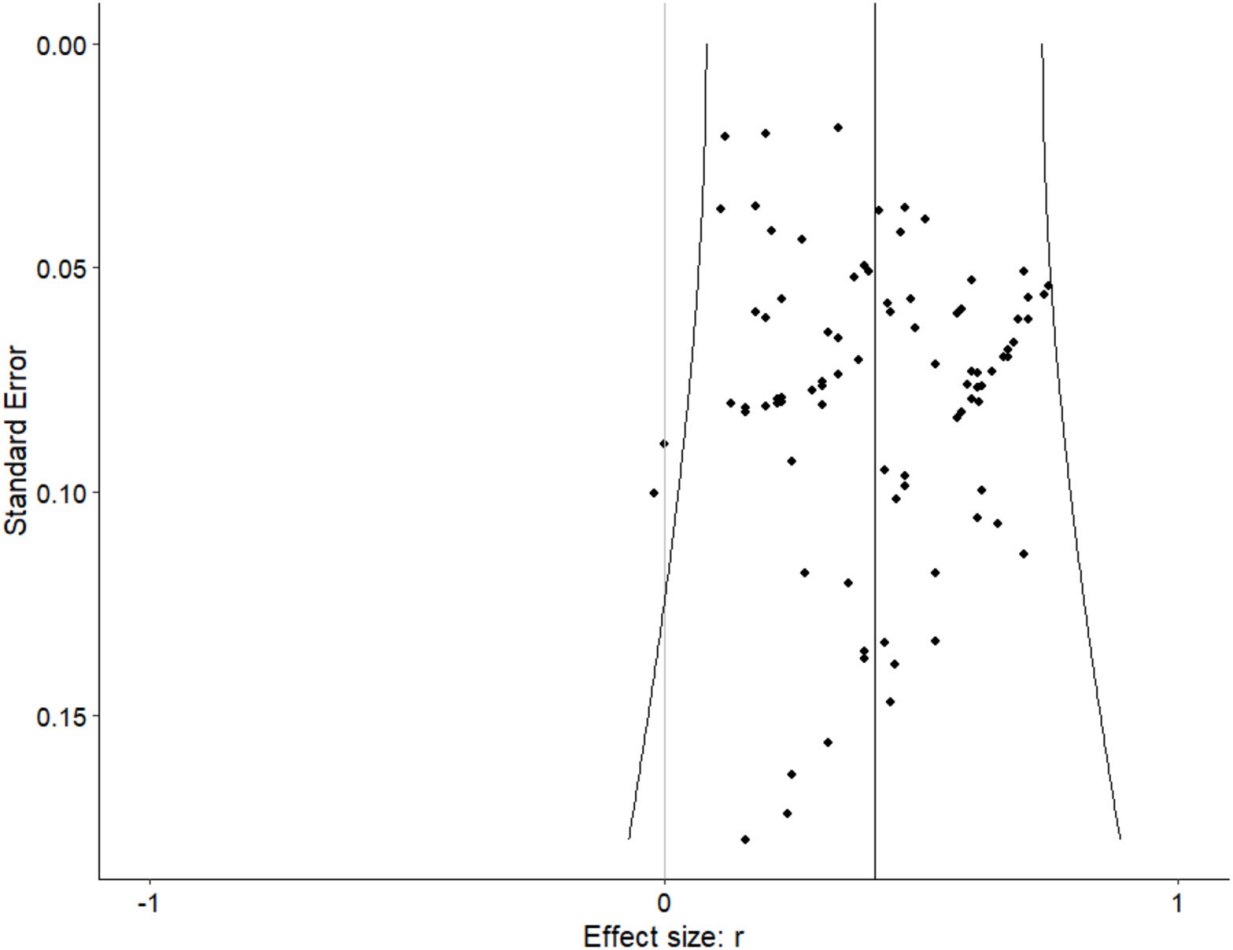
Funnel plot of aggregated effect sizes (*k* = 88) within studies (*s* = 38).

The results of the multilevel meta‐analysis showed a significant medium‐to‐large correlation between interpersonal distress and NR‐MHI, *z* = 0.44, SE = 0.03, 95% CI [0.37, 0.50], *t*(87) = 12.99, *p* < 0.001, *r* = 0.41. The distribution of the effect sizes is presented in the multilevel forest plot at Figure 2. The *I* ^2^ indicated moderate overall heterogeneity of 32.8% at Level 2 (between‐effect size variability) and 57.5% at Level 3 (between‐study variability), Q(87) = 710.38, *p* < 0.001. This indicates that heterogeneity was impacted by particular measures within studies as well as between studies contexts. Inspection of a multilevel funnel plot did not present evidence of publication bias (Figure 3). Following IQR criteria, we did not find outliers within the effects included in the study.

### 4.3 Moderators of Interpersonal Problems and Mental Health Outcomes Association in People With Depression

Following the pre‐registration, we conducted sensitivity analyses testing sample diagnoses or being part of a trial or not as moderators. Results showed no significant moderation effects on the association of interpersonal distress and NR‐MHI by neither sample diagnoses (i.e., being part of a depressive‐specific diagnoses population vs. a population with majority of people with depressive disorder diagnoses), *F*_(1, 86)_ = 0.96, *p* = 0.33, nor by whether subjects were enrolled in a clinical study, *F*_(1, 86)_ = 3.53, *p* = 0.06. Further, a moderation analysis comparing outcome types against depression severity measures (reference category) showed a non‐significant overall moderation effect, *F*_(4, 83)_ = 2.05, *p* = 0.09.

## 5 Discussion

There is a lasting theoretical interest in understanding the potential association between interpersonal distress and depression. This study aimed to test the underlying assumption that interpersonal distress and NR‐MHI are positively associated with each other in depression. We therefore conducted a systematic review and meta‐analysis of available studies that reported such an association to get an evidence‐based estimate of the magnitude and variability of the correlation between interpersonal distress and NR‐MHI across studies. The findings showed that interpersonal distress was significantly related to NR‐MHI in depression. Although meaningful heterogeneity was observed between studies, we did not find significant moderators of the interpersonal distress and NR‐MHI association.

The results of the multilevel meta‐analytic models conducted in this paper established interpersonal distress as a relevant covariate of mental health in depression, presenting a medium‐to‐large effect size. This finding is in line with previous (not meta‐analytic) empirical papers reporting associations between interpersonal distress and specific NR‐MHI in depression such as quality of life (McEvoy et al. 2013), self‐criticism (Dinger et al. 2013) and self‐esteem (Sowislo and Orth 2013) or even overall depressive severity (Barrett and Barber 2007). Additionally, these findings are consistent with systematic reviews and meta‐analyses showing the association of depression with other relational constructs such as social support (Edwards et al. 2020), loneliness (Erzen and Çikrikci 2018) and social skill deficits (Segrin 1990). Considering that all these relational variables might correlate with interpersonal distress, future research would be necessary to determine the specific contribution of interpersonal distress in depression when adjusting for these relational covariates.

Overall, the present meta‐analysis provides the first evidence‐based estimate of the association between depression and interpersonal distress as a basic assumption of the lasting interests in interpersonal theories of depression (e.g., Lewinsohn 1974; Cacioppo et al. 2015; Hopwood et al. 2023). These findings provide further evidence regarding the potential relevance of interpersonal distress in depression, whereas the magnitude of the moderate to strong associations indicates that individuals discriminate between interpersonal distress and depression. Classical interpersonal theories have postulated that interpersonal problems might have an important role in the aetiology and maintenance of depression (Hames et al. 2013). For example, Horowitz and Vitkus (1986) hypothesized that an individual's perception of interpersonal problems partially explains the emergence of psychopathological symptoms such as depression. Regarding depression persistence, Joiner (2000) argued that self‐propagatory processes actively generate a range of interpersonal problems and stressors which serve as robust predictors of prolonged episodes of depression and the emergence of future depressive symptoms. Furthermore, Coyne (1976) postulated that expected interpersonal responses to a depressive style might maintain and intensify depressive symptoms. Contemporaneous models of psychotherapy, such as interpersonal psychotherapy (Weissman et al. 2007), also operate under the assumption that interpersonal stressors, lack of social support and psychological vulnerabilities such as insecure attachment are key factors in predisposing to, triggering and maintaining depression disorders (Ravitz et al. 2011). The results of this study further support the hypothesis of a relevant role of overall interpersonal distress in depression. However, results should be interpreted cautiously considering that not a unidirectional (i.e., causal) effect can be inferred based on these results. In fact, more recent theories have postulated interpersonal pathoplasticity in the course of depression (Cain et al. 2012). This implies that there is a mutual (i.e., not etiological) relationship between maladaptive interpersonal features and depression. Although preliminary evidence has supported interpersonal pathoplasticity in depression, finding various relational styles related to the condition (Cain et al. 2012), future research will need to investigate the role of overall interpersonal distress in the context of this model.

Regardless of the theoretical model to understand the role of interpersonal issues in depression, the empirical results of this meta‐analysis suggest the potential relevance of assessing and targeting interpersonal distress in the treatment of depression. This conclusion is consistent with meta‐analytic studies showing that baseline interpersonal problems predict treatment outcomes in psychotherapy for depressive and anxiety disorders (Gómez Penedo and Flückiger 2023) and with studies reporting that intake interpersonal problems predict differential treatment effects (Constantino et al. 2023; Gomez Penedo et al. 2018). Furthermore, in this same direction, some individual studies have found that outcomes of psychological interventions are related to changes in interpersonal distress (Coyne et al. 2019) and interpersonal style (Gómez Penedo et al. 2019) during treatment. However, meta‐analytic reviews may need to be performed to provide more sound evidence regarding the role of interpersonal distress as a putative mechanism of change in psychological treatment.

This study has several limitations that need to be addressed in future research. First, in this study, we only focused on the general interpersonal distress, whereas there are other more specific indicators of interpersonal dysfunction/problems grounded on interpersonal dimensions such as agency and communion (Luyten et al. 2010; Quilty et al. 2013; Wiltink et al. 2016) or specific subtypes of interpersonal problems, such as (being too) domineering, intrusive, overly nurturant, exploitable, nonassertive, socially inhibited, cold and vindictive (Dinger et al. 2013; Luyten et al. 2010; Schauenburg et al. 2000). Future studies would need to make finer‐grained analyses to determine how different types or clusters of interpersonal difficulties can be related to non‐relational mental health outcomes in depression. In this same vein, when defining interpersonal distress, we relied exclusively on self‐reported measures. Future studies would need to explore how other interpersonal distress indicators and from different perspectives are associated with mental health outcomes in depression. Furthermore, in this meta‐analysis, we observed significant heterogeneity across the different studies included in the systematic search, regarding their methods and the way they reported their results. Future studies will benefit from using approaches such as the individual patient data meta‐analysis, which provides a way of aggregating and synthesizing data from different studies with various designs into a meta‐analytic framework. In addition, in the current study, we tested a series of limited moderators that were not significant. Nonetheless, the significant variability observed in the multilevel models suggests the possibility of the existence of other variables not explored in this study that might be meaningfully moderating this effect.

Besides these potential limitations, our study is the first to provide an estimation of the association between interpersonal distress and mental health outcomes in depression. The results highlight interpersonal distress as a significant covariate to be taken into account and to further explore in people suffering from depressive disorders.

## Conflicts of Interest

The authors declare no conflicts of interest.

## Data Availability

The data supporting the findings of this study are available from the corresponding author upon reasonable request.
